# Neuromuscular electrical stimulation and biofeedback therapy may improve endometrial growth for patients with thin endometrium during frozen-thawed embryo transfer: A preliminary report

**DOI:** 10.1186/1477-7827-9-122

**Published:** 2011-08-25

**Authors:** Madafeiton MA Bodombossou-Djobo, Chengyu Zheng, Shaoqing Chen, Dongzi Yang

**Affiliations:** 1 Department of Obstetrics and Gynecology, Reproductive Center, Memorial Hospital of Sun Yat-Sen University, Guangzhou, Guangdong, China

## Abstract

**Background:**

To investigate the effect of pelvic floor Neuromuscular Electrical Stimulation (NMES) Therapy in improving endometrial thickness in women with thin endometrium.

**Methods:**

41 patients undergoing assisted reproduction with a thin endometrium (less than or equal to7 mm) were recruited and advised to go for a pelvic floor NMES in frozen-thawed embryo transfer cycle. PHENIX Neuromuscular Electrical Stimulation Therapy System was used according to the manufacturer's recommended protocol for 20 to 30 minutes of intermittent vaginal electrical stimulation on the treatment days.

**Results:**

A total of 20 and 21 were included in the NMES and non-NMES groups respectively. 12 out of 20 (60%) patients developed endometrial thickness equal to or more than 8 mm after the NMES therapy, which was the primary outcome. The mean thickness of endometrium before and after was respectively 5.60 mm (0.82 mm) and 7.93 mm (1.42 mm) in the therapy group versus 5.50 mm (1.00) and 6.78 mm (0.47) in the control group; the difference was statistically significant (P = 0.002). There was higher pregnancy rate in the NMES group (42% versus 35%) but the difference was not statistically significant.

**Conclusion:**

Neuromuscular Electrical stimulation therapy may be effective for the patients with a thin endometrium. Further studies are needed to investigate its effectiveness.

## Background

By the time of ovulation, many assisted reproductive technologies (ART) practitioners like to see the endometrium at least 8 mm thick. An adequate endometrium thickness and appearance play a key role in the implantation of embryo and achievement of pregnancy [1-3]. There is no officially accepted definition of "thin lining". The commonly accepted cut off would be less than 8 mm on the day of LH surge or HCG administration day [4]. Thin endometrium at the time of ovulation can be a concern and has been demonstrated to be an important factor in implantation failure [5]. The exact mechanism of implantation failure is unknown but might be related to an overall poor intrauterine environment. Factors affecting the growth of endometrium are still not well understood, but recently many researchers have focused on the angiogenesis and vascularization within the endometrium and found that a poor uterine receptivity in women with thin endometrium may be due to the impairment of blood flow impedance through the endometrium [6-9]. Recent studies have focused on different treatment modalities which include exogenous estrogen [10], low-dose aspirin [11-13], vaginal sildenafil citrate [14], pentoxifylline, vitamin E, L-arginine [9,15,16], gonadotropin-releasing hormone agonist [17], electroacupuncture [8] and cytokine (granulocyte colony stimulation factor) [18] for the management of women with thin endometrium with an aim to increase the implantation and pregnancy rates in ART cycles. Although there are many studies concerning different managements of women with thin endometrium to modulate uterine artery blood flow and improve endometrial thickness, there is no agreement in the literature about a consensus treatment.

Up to date, no study has evaluated the use of Pelvic floor neuromuscular electrical stimulation (NMES) in reproductive medicine. NMES has been used in urogynecology and obstetrics during pregnancy and postpartum period. There is a strong evidence for its effectiveness for treatment of stress urinary incontinence (SUI), pelvic pain, sexual dysfunction, low back pain and constipation in clinical practice [19]. NMES is the application of electrical stimulation to a group of muscles through electrodes placed on the skin. It is the elicitation of muscle contraction using electric impulses. The impulses are generated by a device and delivered through electrodes on the skin in direct proximity to the muscles to be stimulated. Long-term data reported that the rate of patients cured or patient's conditions improved from electrical stimulation ranged from 54-77% [20]. The purpose of this study was to investigate whether thin endometrium can be improved by pelvic floor NMES.

## Methods

The study was reviewed and approved by the Ethical Review Board of the Memorial Hospital of Sun Yat-Sen University. Written informed consent was obtained from all patients.

### Subjects and study design

This study was an observational and prospective study. It was carried out in the reproductive center of the Memorial Hospital of Sun Yat-Sen University (Guangzhou, China) from December 2009 to April 2011. Women were eligible for the study if they were 20 to 39 years old, if they were recorded at least 2 previous assisted reproductive treatment cycle failures in which the optimum endometrium thickness was ≤ 7 mm and were undergoing frozen-thawed embryo transfer in hormone replacement therapy (HRT) cycle.

Women were excluded from participation if they had a medical history that included pelvic cancer, severe endometriosis, Asherman's syndrome, adenomyosis, or congenital uterine anomaly, use of an intrauterine device, vaginitis, neurologic disorders, systemic diseases, hypertension or diabetes mellitus. Exclusion based on physical examination by the nurse practitioner (the physical therapist) occurred if women had vaginal wall prolapse, skin breakdown around the peri-anal region, rectal or vaginal bleeding, or absolute contraindications for pelvic neuromuscular electrical stimulation such as complete denervation of the pelvic floor (will not respond), dementia, cardiac pacemaker, unstable or serious cardiac arrhythmia, unstable seizure disorder, pelvic pain and painful hemorrhoids.

A total of 41 women with a history of infertility and who had a thin endometrial (endometrial thickness ≤7 mm in the late follicular phase) in their previous treatment cycles were recruited into this study. This threshold was chosen based on studies in the literature reported reduced implantation and pregnancy rates when endometrial thickness was 7 mm and less[5,21,22]. All the women were advised to go for a pelvic floor NMES therapy from day9 or day10 for 3 to 4 times consecutively (qd for 20-30 minutes). 20 women accepted to try NMES. The choice of the patients not to go for NMES did not affect their further treatment.

All the women received previously a standard regime of the GnRH agonist with the use of (Triptorelin) and gonadotropins (Gonal-F). Human chorionic gonadotropin (10.000 IU) was administered intramuscularly when lead follicles were ≥ 18 mm in diameter, and there were at least three follicles 16 mm in diameter (IVF/ICSI). 30-36 h after injections of HCG, oocyte was retrieved guided by transvaginal ultrasound. They had a fresh embryo transfer cycle failure and at least one frozen-thawed embryo transfer (FET) cycles on natural cycle, ovulation induction cycle or on artificial cycle without success.

### Endometrial preparation in HRT cycle

Estradiol valerate (E2) 2 mg/day (Progynova; Bayer Schering Pharma, France) was commenced orally on day 3 of menstruation cycle without prior pituitary suppression and continued for 5 days. After 5 days (day8), the dose of progynova was changed to 2 mg bid for 3 days. On day10 of menstruation cycle, endometrial thickness was assessed by ultrasound. If the endometrial thickness was ≤8 mm, the dose of progynova was increased to 3 mg bid for 3-5 days, after which the endometrial thickness was evaluated for the second time. If the endometrial thickness was still ≤8 mm, the dose was increased to 5 mg bid until the endometrial thickness reached 8 mm. If the endometrium thickness reached 8 mm, the luteal phase support was started (D0) by providing progesterone (60 mg daily by intramuscular injection or 90 mg of progesterone sustained-release vaginal gel, daily for 17 days) to all patients. If the endometrial thickness failed to reach 7 mm after 20 days of progynova use, the cycle was usually cancelled and restarted in a new protocol (natural cycle or ovulation induction cycle), but the final decision was given to patient to go forward for the embryo transfer or cancel the cycle. All the embryos transfer were performed on D3 (72 h after beginning progesterone supplementation).

### Pelvic Floor Neuromuscular Electrical Stimulation therapy

#### Guide for pelvic floor muscles contraction

The women were told to lie down in the supine position, lithotomy position, on an examination bench with the legs extended. The nurse practitioner (physical therapist) examined all participants prior to therapy. The physical therapist instructed them how to contract and relax the pelvic floor muscles. A digital vaginal exam was used to help the physical therapist make sure whether the patient successfully contracted the right muscles or not and determine the degree of muscles strength by contraction or compression of examiner's fingers. Each contraction should involve a concentrated effort to get maximum tightening. Patients were instructed to contract only the pelvic floor muscles. If they feel their abdomen, thighs or buttocks tightening then they were asked to relax and aim just for the pelvic muscles by using a less intense muscle contraction. The women were encouraged through detailed and explicit instructions and directions by the physical therapist.

#### Therapy posture

The patient still in supine position with one cushion under the head and the legs opened up, both of the legs forming 40° approximately. Two other cushions of about 5-10 cm height were inserted under the knees to prevent the thigh muscles from participating in contraction of pelvic muscles. An ointment (electrical conductance gel) was applied to the tip of the probe before its insertion into the vagina. The probe was inserted to about 6-8 cm deep. The vaginal probe was fixed on the probe box, situated between the two cushions, to avoid squeezing it out of the vagina. All the women had their own vaginal probe (Guangzhou Shanshan Medical Apparatus and Instruments Industry CO. LTD ZL2005201203870, China). Adhesive electrodes were placed on the skin outside the vagina (on the women's thigh). Vaginal probe was kept in vagina and electrical stimulation was initiated.

### Neuromuscular electrical stimulation and biofeedback therapy

PHENIX Neuromuscular Electrical Stimulation Therapy System USB 4 (Guangzhou Shanshan medical apparatus and instruments industry CO. LTD, Guangzhou, China) was used according to the manufacturer's recommended protocol for 20 to 30 minutes of intermittent vaginal electrical stimulation on the treatment days. Selected parameters included biphasic intermittent current, frequency 40 Hz, pulse width 250 us, and current intensity between 0-120 mA with individually adapted on-off (duty) cycles on the basis of each woman's ability to hold a voluntary contraction. On time ranged from 3 seconds to 10 seconds, and off time from 0 second to 30 seconds. For biofeedback therapy, computer graphs were used to help them locate the pelvic muscles. All patients were encouraged to tolerate as high intensity as possible to get a contraction.

### Kegel Maneuvers

After the first therapy, the women were instructed to practice exercises at home. The exercise program consisted on to hold each contraction for 1 second and then to relax the muscles for 5 seconds. This sequence was repeated continuously for 15 minutes. The women were told to do the exercise in lying position so that there is little stress on the muscles. Two training repetitions were performed in the presence of the physical therapist.

### Endometrial evaluation

Endometrial thickness was measured at its thickest part in the longitudinal axis of the uterus before (day9 or day 10) and after NMES therapy (D0, the start of progesterone supplementation for FET) in the two groups. The endometrial pattern visualized was designated as a multilayered or a non-multilayered endometrium. The measurements were performed by one investigator (C.Z) using a computerized vaginal ultrasound with an integrated pulsed Doppler vaginal scanner (Aloka Prosound SSD-3500., Japan). CZ was blind to the conditions of the patients.

### Statistical analysis

Mean and standard deviation were used for description of variables. A student's t test was used to compare the difference between groups. Comparison of before and after treatment endometrial thickness in the NMES group was performed using Non-parametric Wilcoxon test. Statistical comparison for percentage was carried out by chi-square. All the statistical analyses were carried out using SPSS software (Statistical Package for Social Sciences, SPSS Inc, Chicago, IL, USA) version 16.00. A p value < 0.05 (two tailed) was considered to be statistically significant.

## Results

### General characteristics of the study population

The general characteristics of the study subjects are shown in Table 1. A total of 20 and 21 women were included in the NMES and nonNMES treatment groups, respectively. Their mean age was 30.10 ± 3.97 years (range 24-39). Their mean body mass index was 20.44 ± 2.55 kg/m^2 ^(range 16.60-27.20). The women in this study had mixed diagnosis. 24/41(58.5%) had primary infertility. 19 women out of 41 were diagnosed polycystic ovary syndrome (PCOS). 31 had tubal occlusion, and 29 with male factor. The mean age and endometrial thickness at baseline (day9) of the two groups showed non-significant difference (29.75 ± 3.95 years versus 30.62 ± 4.54 years; 5.60 ± 0.82 mm versus 5.50 ± 1.00 mm) (Table 1)

**Table 1:** Comparison of Age, BMI, Waist, Baseline hormones profile, Endometrial Thickness, and Pregnancy Rate in NMES and nonNMES Groups

Parameters	NMES GROUP (n = 20)	nonNMES Group (n = 21)	P value
Age (years)	29.75 +/- 3.95	30.62 +/- 4.54	0.516
BMI (Kg/m^2^)	20.44 +/- 1.94	20.45 +/- 3.10	0.987
Waist (cm)	72.03 +/- 4.94	71.10 +/- 7.75	0.663
bFSH (IU/ml)	7.03 +/- 3.09	8.20 +/- 3.69	0.296
bLH (IU/ml)	9.70 +/- 7.68	6.72 +/- 5.50	0.174
bE_2 _(ng/ml)	54.75 +/- 47.34	64.16 +/- 51.90	0.563
bT (nmol/l)	1.75 +/- 1.01	1.71 +/- 0.67	0.874
Infertility			
Primary	11(55%)	13(61.9%)	
Duration of infertility (years)	3.85 +/- 2.78	5.40 +/- 3.22	0.111
Tubal factor	14(70%)	17(81%)	
Male factor	10(50%)	19(90%)	
PCOS	12(60%)	7(33.3%)	
Endometrium thickness at day10 (mm)	5.60 +/- 0.82	5.50 +/- 1.00	0.731
Endometrium thickness at Day 0 (mm)	7.93 +/- 1.42	6.78 +/- 0.47	0.002
Pregnancy rate (%)	8(42%)	7(35%)	0.748
Number of Embryos transferred	1.85 +/- 0.49	2.43 +/- 0.75	0.006

Data are shown as mean +/- standard deviation and number (percentage). Day 0: start of progesterone supplementation in HRT cycle. HRT: Hormone replacement therapy. PCOS: Polycystic ovarian syndrome. P < 0.05 was statistically significant.

### Primary outcome

After the NMES therapy, the endometrium became thickened in the NMES group (7.93 ± 1.42 versus 6.78 ± 0.47; P = 0.002) which was statistically significant. The majority of patients in the NMES group showed an improvement in the endometrial thickness. 12/20(60%) patients developed endometrial thickness equal to or more than 8 mm after NMES therapy, which was the primary outcome. 3 out of 20 did not respond to the therapy(no change was observed in their endometrium thickness during the cycle).

### Secondary outcome

"Regarding the clinical pregnancy rate, 42% (8/19) and 35% (7/20) conceived in the NMES group and nonNMES group respectively. There was no significant difference between the NMES group and nonNMES group (Table 1). The mean thickness endometrium in women who conceived was 7.79 ± 1.13 mm, the one in non-pregnant women 7.18 ± 1.18 and the women who conceived were younger than the ones who did not (28.67 ± 2.44 versus 30.92 ± 4.94) but the differences were not statistically significant (Table 2).

**Table 2:** Comparison of Age, BMI, Waist, Baseline hormones profile, and Endometrial Thickness in Pregnant and nonPregnant Groups

Parameters	Pregnant women (n = 15)	nonPregnant women (n = 26)	P value
Age (years)	28.67 +/- 2.44	30.92 +/- 4.94	0.067
BMI (Kg/m^2^)	20.85 +/- 2.53	19.78 +/- 2.13	0.170
Waist (cm)	73.39 +/- 4.47	69.27 +/- 6.09	0.036
bFSH (IU/ml)	7.16 +/- 3.60	7.88 +/- 3.49	0.554
bLH (IU/ml)	11.53 +/- 9.22	6.44 +/- 4.28	0.069
bE2 (ng/ml)	59.49 +/- 47.64	62.84 +/- 52.16	0.847
bT (nmol/l)	1.85 +/- 0.46	1.68 +/-1.03	0.499
Duration of infertility(years)	3.71 +/- 2.40	5.13 +/- 3.25	0.166
Endometrium thickness at day10 (mm)	5.64 +/- 0.93	5.46 +/- 0.93	0.559
Endometrium thickness at Day 0 (mm)	7.79 +/- 1.13	7.18 +/- 1.18	0.120
Number of Embryos transferred	2.2 +/- 0.41	2.29 +/- 0.55	0.583

Data are shown as mean +/- standard deviation and number (percentage). Day 0: start of progesterone supplementation in HRT cycle. P < 0.05 was statistically significant.

2 women (1 in the NMES group and 1 in the nonNMES group) had embryo transfer cancelled because of a poor embryo quality.

## Discussion

NMES is the application of electrical stimulation to a group of muscles through electrodes placed on the skin. It is the elicitation of muscle contraction using electric impulses. The impulses are generated by a device and delivered through electrodes on the skin in direct proximity to the muscles to be stimulated. The impulses mimic the action potential coming from the central nervous system, causing the muscles to contract. It was primarily used by physical therapists to help restore function to injured muscles. Now the utilization of NMES is a routine practice in urology (incontinence) and in gynecology & obstetrics (pelvic pain, dyspareunia) all over the world [23-25]. The effectiveness of pelvic-floor strengthening exercise and electrical stimulation in the management of SUI is well established [25,26]. To the best of our knowledge, this is the first study investigating physical therapy, pelvic floor NMES to improve endometrial growth in women with thin endometrium. We have shown that NMES could be an effective option to manage women with thin endometrium. All of the patients reported in this study had a least two prior failed ART treatment and none of them had previously shown an endometrium thickness >7 mm. Our study showed that pelvic floor NMES significantly enhances endometrial thickness in patients with thin endometrium. Uterine blood flow has been showed to be an important factor for endometrial growth. It is therefore likely that NMES corrects the impairment of uterine blood flow impedance. Most of the women who received NMES therapy in this study showed a remarkable improvement in the endometrium thickness.

Little information is available regarding the exact mechanisms by which NMES exerts its function on the process of angiogenesis and vascularization in the endometrium. However, based on the data in the literature on the importance of angiogenesis and vascularization for a receptive uterine, we believe that by stimulating uterine smooth muscle to repeated contraction and relaxation, there is an increased in blood supply towards the whole endometrial and the sub-endometrial regions that leads to peripheral tissue trophicity. NMES provides a passive contraction that increases awareness of pelvic floor muscles contractions in general. The etiology of thin endometrium is complex, multifactorial and remains unclear. High blood flow impedance of uterine radial arteries and decreased in vascular endothelial growth factor (VEGF) expression are associated with poor endometrial growth [21]. The inadequate endometrial thickness might be due also to either fewer estrogen receptors or the desensitization of estrogen receptors. The important point for treatment in case of blood impedance is starting the treatment at the first day of the menstrual cycle. However, NMES was started from day 9 or day10 in the treatment cycle in the present study, to make sure that menstruation had stopped and to avoid excessive bleeding if done during the menstruation period; vaginal bleeding or any internal or external sign of hemorrhage being one of the contraindications for NMES therapy.

With this method, the endometrial thickness was significantly increased and we achieved a pregnancy rate as high as 42%(8/19). Our results are with agreement to those that reported a positive correlation between endometrial thickness and pregnancy rate[3,5,21,22].

In this study, only 3 women did not respond at all to NMES. The uniqueness of this study is that it demonstrated a speedy and significant increase in endometrial thickness. NMES was performed only 3 to 4 times for 20-30 min in the subsequent cycle (once per day consecutively). The number or amount of time of therapy needed to show improvement varies from person to person. NMES therapy was stopped as soon as the endometrial thickness reached 8 mm. Four times therapy was the maximum we prescribed to the women in this study. Other benefits include NMES being safe, it is also a noninvasive technique and it appears as a promising alternative for managing patients with thin endometrium. No known serious adverse effects have been reported by the subjects.

## Conclusions

Pelvic floor NMES showed a positive effect on the endometrium thickness and the pregnancy rate, but there may be too few subjects in the present study to draw a firm conclusion. Further studies are doubtless needed in this area to investigate this issue.

## Competing interests

The authors declare that they have no competing interests.

## Authors' contributions

MMABD carried out the acquisition of data, analysis and interpretation of data and writing of the manuscript. CZ has been involved in the ultrasound examination and in drafting the manuscript and revising it critically for important intellectual content. SC, physical therapist, carried out the pelvic floor neuromuscular electrical stimulation therapy. DY conceived of the study, and participated in its design and coordination, helped to draft the manuscript and have given final approval of the version to be published. All authors have read and approved the final version of the manuscript.
